# Testicular ischemia in deficiency of adenosine deaminase 2 (DADA2)

**DOI:** 10.1186/s12969-019-0334-5

**Published:** 2019-07-10

**Authors:** Katherine Clarke, Cathy Campbell, Ebun Omoyinmi, Ying Hong, Muthana Al Obaidi, Neil Sebire, Paul A. Brogan

**Affiliations:** 1 Department of Paediatric Rheumatology, Great Ormond Street Hospital NHS Foundation Trust, London, UK; 2 Infection Inflammation and Rheumatology Section, University College London Great Ormond Street Institute of Child Health, London, UK; 3 Department of Histopathology, Great Ormond Street Hospital NHS Foundation Trust, London, UK

**Keywords:** Deficiency of adenosine deaminase 2, DADA2, Testicular infarction, Vasculitis, Anti-tumour necrosis factor alpha, Aspirin, Biomarker, Child

## Abstract

**Background:**

Deficiency of adenosine deaminase 2 (DADA2) is a rare autosomal recessive autoinflammatory condition. Recognised features include vasculitis predominantly affecting medium sized vessels, livedoid skin rash, central and peripheral nervous system involvement, variable degrees of immunodeficiency, and marrow failure, amongst other clinical presentations. We present the case of a six year old male with DADA2 who presented with acute testicular ischaemia secondary to vasculitis, the first such description in DADA2.

**Case presentation:**

A six year old male presented acute right-sided testicular pain. His history included transient infantile neutropenia, resolved hepatosplenomegaly, and longstanding livedo racemosa, leading to screening and confirmation of DADA2 caused by homozygous c.139G > C (p.G47R) mutation of *ADA2.* As his only clinical feature was that of mild livedo racemosa with normal laboratory parameters at diagnosis, he was being actively monitored prior to starting any treatment. At a routine clinic follow-up a 24 h history of testicular pain was noted on systems review. He was afebrile, and his only physical signs were that of moderate livedo racemosa, and tenderness of the right testicle. Laboratory parameters revealed C-reactive protein (CRP) 8 mg/L (reference range [RR] < 20 mg/L); erythrocyte sedimentation rate (ESR) 28 mm/hr. (RR < 10); and serum amyloid A (SAA)5 mg/L (RR < 10). Ultrasound-scan of the scrotum revealed significantly reduced perfusion of the right testes, without torsion. Surgical scrotal exploration confirmed testicular ischaemia without torsion. Histology demonstrated ischaemic seminiferous tubules with intervening haemorrhage and acute inflammatory cells, consistent with vasculitis of the testis as the cause. He was treated with high dose intravenous methyl-prednisolone followed by a weaning course of oral prednisolone, and subcutaneous adalimumab (anti-tumour necrosis factor alpha, anti-TNFα). Repeat ultrasound-scan 3 weeks later revealed good testicular perfusion, with a small area of focal infarction. At last follow-up (11 months post-event) he remained asymptomatic, on treatment with adalimumab.

**Conclusion:**

The phenotype of DADA2 continues to expand, and we add testicular infarction to the features of DADA2. CRP and SAA cannot be relied on as reliable biomarkers to predict tissue ischaemia and hence who to target for anti-TNFα therapy in DADA2, since these remained steadfastly normal before, during, and after testicular infarction in this case.

## Background

Deficiency of adenosine deaminase 2 (DADA2) is a recently described recessive genetic autoinflammatory disease [1, 2], with variable clinical penetration and severity, even in families with individuals sharing the same mutation [3]. Commonly encountered clinical features include systemic inflammation, recurrent fevers, various vasculitic features resembling polyarteritis nodosa (PAN) including livedo racemosa, vasculitic ulcers, and various other cutaneous vasculitic phenomena; and systemic vasculitis affecting virtually any organ in the body but particularly the neurological system with propensity to strokes (both lacunar and haemorrhagic) [1, 2, 4, 5], and also the peripheral nervous system [3]. DADA2 is also associated with varying degrees of immunodeficiency (immunoglobulin deficiency) [3], marrow failure, ranging from single cell lines affected (pure red cell aplasia mimicking Diamond-Blackfan anaemia; or neutropenia), through to complete marrow failure [4, 6]. Lymphoproliferation mimicking Castleman’s disease has also been described [7]. Whilst designated an autoinflammatory disease, autoimmunity has also been described in DADA2 [8]. Asymptomatic (sometimes referred to as “pre-symptomatic”) adults with DADA2 detected on genetic screening of index cases are also recognised, further emphasising the lack of genotype/ phenotype correlation [3].

Bi-allelic loss-of-function mutation in *ADA2* located on chromosome 22q11.1 results in loss of enzyme activity of adenosine deaminase 2 (ADA2) [1, 2], a dimeric 57 kDa extracellular isoform of adenosine deaminase 1 (ADA1: deficiency of which causes severe combined immunodeficiency) [1, 9, 10]. Despite its name, the adenosine deaminase enzyme activity of ADA2 is 100-fold lower than ADA1, although this rises at higher temperatures and acid pH [9]. Whilst the function of ADA2 is still poorly understood, this is an area of intense ongoing research. The current consensus view is that although the receptor for ADA2 has not yet been identified [11], the main role of ADA2 is probably that of a growth factor influencing the haematopoietic system, a function which is possibly independent of its adenosine deaminase enzymatic activity [1, 9]. Patients with DADA2 demonstrate skewing towards pro-inflammatory M1 macrophage populations, with relative paucity of anti-inflammatory M2 macrophages [1]. These M1 macrophages produce several pro-inflammatory cytokines, and interact with endothelium to initiate vasculitis [1, 10].

There has never been a randomised, controlled therapeutic clinical trial for the treatment of DADA2. Despite that, there is a very strong prevalent clinical experience suggesting therapeutic benefit for TNF blockade (anti-TNF; often combined with corticosteroids at therapy initiation), with no reports of cardiovascular events occurring in patients treated with adequate doses of anti-TNF [2–4, 12]. Although almost certainly a poly-cytokine disease, the same is not true of IL-6 blockade, since patients have been described with vasculitic events (including stroke) whilst on tocilizumab [3]. Likewise, interleukin-1 blockade anecdotally is not effective for inflammatory sequelae of DADA2, although canakinumab might be effective for AA amyloidosis secondary to DADA2 [13]. Allogeneic haematopoietic stem cell transplantation (HSCT) can be effective for selected patients with marrow failure and/ or severe immunodeficiency; vasculitic and other autoinflammatory features also respond completely to HSCT [14].

We present the first description of vasculitic testicular infarction in a child with DADA2, thus expanding the phenotype even further, and highlight controversies and current unmet needs for this newly described genetic autoinflammatory disease.

## Case presentation

A six year old Indian male born to non-consanguineous parents presented for routine outpatient review for monitoring of deficiency of adenosine deaminase 2 (DADA2). On systems review, he had a 24-h history of nausea and vomiting, followed by acute, moderate right-sided testicular pain, without fever.

His past medical history included an isolated episode of transient neutropenia as an infant, in association with hepatosplenomegaly which was considered at the time to be due to intercurrent viral infection. These features resolved completely, but at the age of 5 years he was referred to our centre with lower limb rash considered to be cutaneous PAN. Examination revealed typical livedo racemosa mainly involving the lower limbs, with no other abnormalities detected on full examination of all systems. Consequently, genetic screening of *ADA2* and assessment of serum ADA2 enzyme activity was undertaken. These revealed homozygous c.139G > C mutation (p.G47R) of *ADA2*, and absent (i.e. undetectable) serum ADA2 enzyme activity, detected as part of routine care, as previously described by our group [3], confirming the diagnosis of DADA2. His parents were heterozygous for the same mutation; a 3 year old female sibling was bi-allelic wild type for *ADA2*; and there was no family history of vasculitis, autoimmunity or immunodeficiency. Investigations performed prior to the acute presentation as part of general workup for DADA2 revealed: normal complete blood count (including normal differential white cell counts); normal renal and liver function; serum amyloid A (SAA) 8.2 mg/L (reference range [RR] < 10 mg/L); C-reactive protein (CRP) < 5 mg/L (RR < 20 mg/L); erythrocyte sedimentation rate (ESR) < 10 mm/hour (RR < 10 mm/hour); normal coagulation screen; negative viral hepatitis screen; normal immunoglobulin G, A, and M levels; negative screening for coeliac disease; negative autoantibody screening (Anti-nuclear antibodies (ANA) double-stranded deoxyribonucleic acid (DNA), ANCA (Anti-neutrophil cytoplasmic antibodies) rheumatoid factor, and thyroid peroxidase antibodies); and normal brain and brainstem magnetic resonance imaging, and magnetic resonance angiography. Since his only clinical feature was mild cutaneous involvement, and he was otherwise completely asymptomatic and with completely normal laboratory and imaging workup, he was kept under close surveillance without treatment, since it is known that DADA2 can vary considerably in clinical severity, with adult asymptomatic cases described previously [3].

**Fig. 1:**
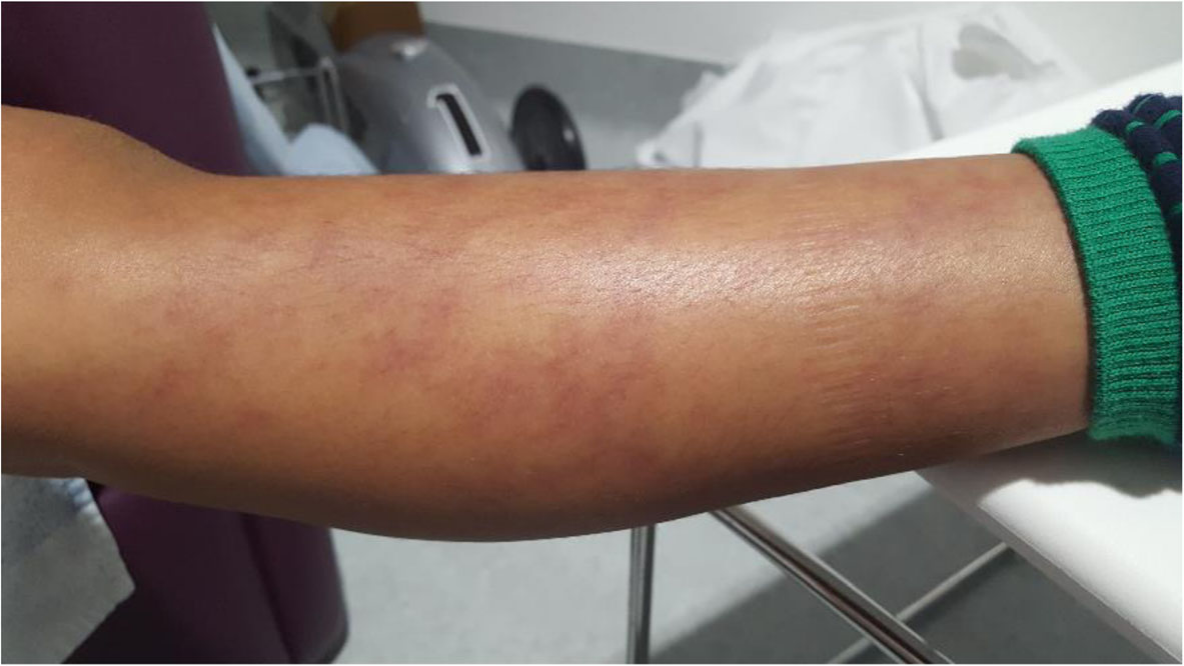
Livedo racemosa noted on both lower limbs

**Fig. 2:**
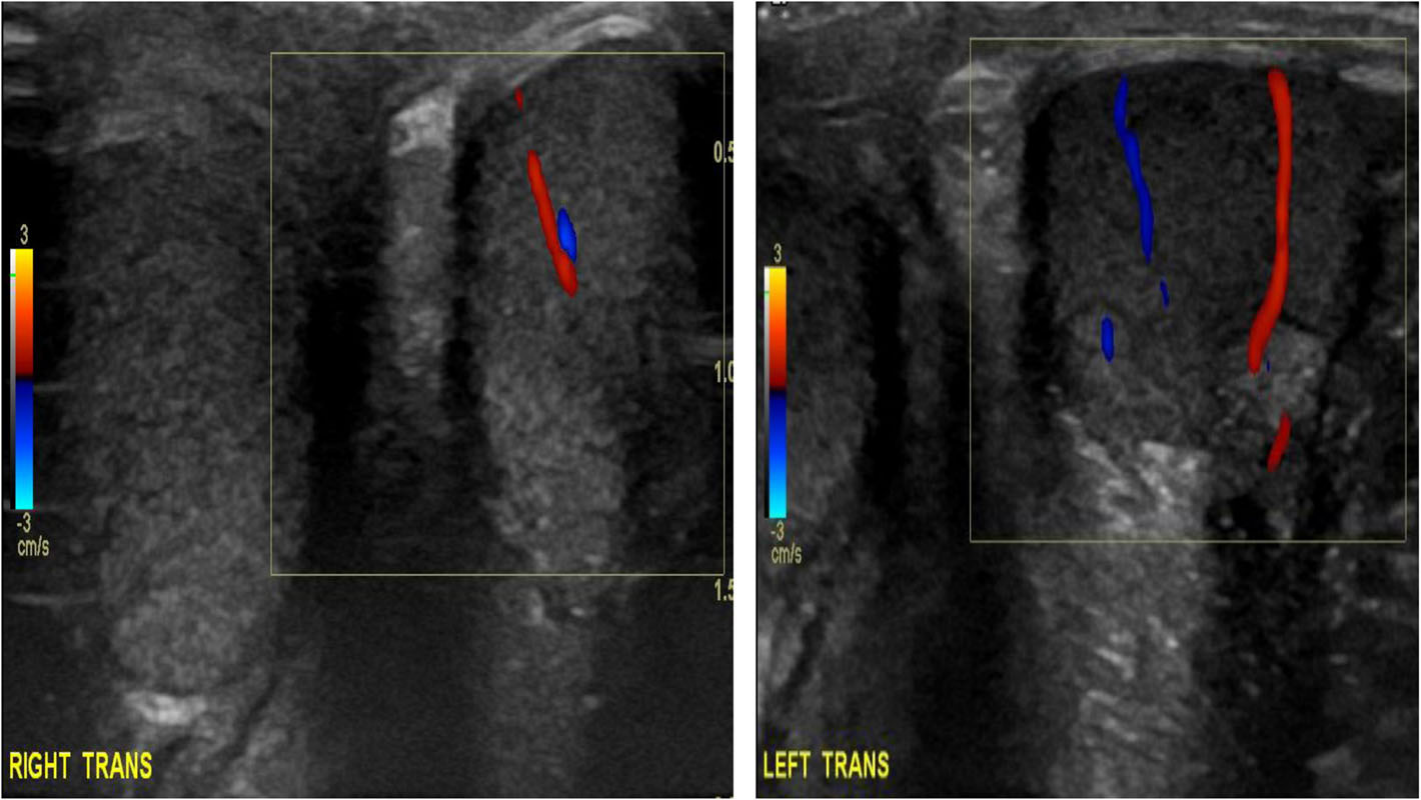
Doppler study of both testes (transverse views) showing globally reduced (but not absent) perfusion in the right testes compared to the left

**Fig. 3:**
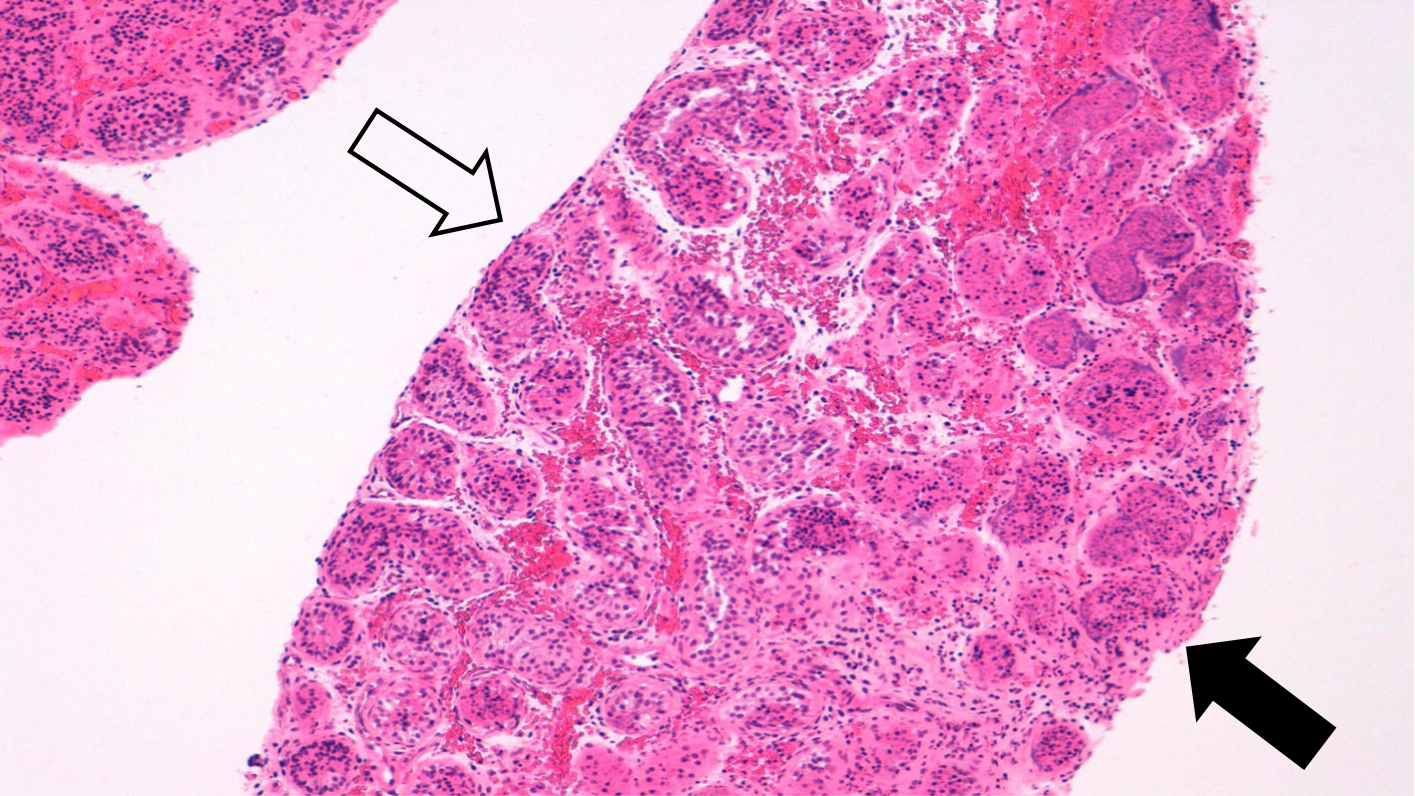
Photomicrograph of testicular biopsy showing patchy areas of tubular necrosis with loss of cellular and nuclear detail and nuclear ‘smudging’ (Right side) with more normal, viable tubules (Left side); haematoxylin and eosin, original magnification × 100

**Fig. 4:**
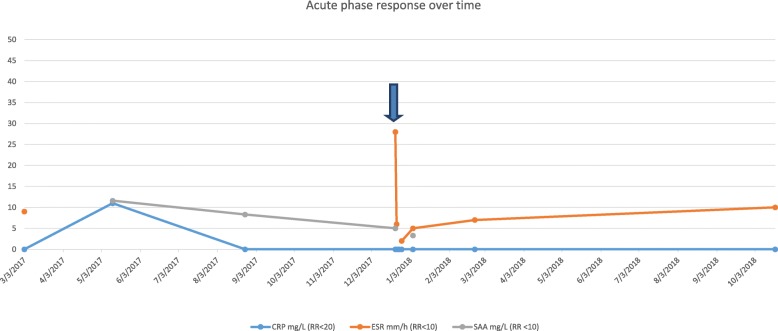
Acute phase markers before, during, and after acute testicular infarction in DADA2. Acute phase responses were completely normal prior to testicular infarction. C-reactive protein (CRP) and serum amyloid A (SAA) remained within the normal range throughout the episode and follow-up. Erythrocyte sedimentation rate (ESR) was normal (measured only once prior to the acute presentation), but was transiently modestly elevated on the day of tissue infarction (arrowed), remaining normal at follow-up (see main text for treatment received)

At the acute presentation, he was afebrile and haemodynamically stable. Systemic examination including full neurological examination was normal except for livedo racemosa on his lower limbs (Fig. 1). He was also noted to have a mildly swollen and tender right testicle, but without typical clinical features of torsion. Repeat laboratory workup revealed a normal full blood count (haemoglobin 125 g/L; total white cell count 6.56 X10^9^/L [RR 5–15]; platelets 306 X10^9^/L [RR150–450]); normal liver, renal and coagulation profile. ESR was mildly elevated at 28 mm/hr. [RR < 10]; CRP of 17 mg/L [RR < 20]; and SAA 5 mg/L (RR < 10). Screening for antiphospholipid syndrome was negative for lupus anticoagulant (normal direct Russell Viper venom test; and negative anticardiolipin IgG autoantibodies). Ultrasound-scan (USS) of the testes showed significantly reduced perfusion of the right testes compared to the left (Fig. 2), with no suspicion of torsion. He underwent an urgent surgical exploration and testicular biopsy. There was no evidence of testicular torsion found intraoperatively. Histology showed ischaemic seminiferous tubules with intervening haemorrhage and acute inflammatory cells which was consistent with an underlying vasculitic process, although no vessels were visible in the specimen to confirm that definitively (Fig. 3). Post-operatively, he was pulsed with intravenous methyl-prednisolone (30 mg per kilogram for 3 consecutive days); followed by 2 mg per kilogram of daily oral prednisolone tapering off over 6 weeks; adalimumab 20 mg subcutaneously every 2 weeks (dose-banded, based on weight of 27.4Kg); and anti-aggregant dose of aspirin, 75 mg daily. He recovered well from the surgery and was discharged on post-operative day 3. Repeat USS at three weeks showed good perfusion of the testicular parenchyma on the right, with a small area of focal infarction. At last follow-up (11 months after the acute presentation) he remains well on 40 mg of adalimumab every 2 weeks (weight now 32 kg), and daily aspirin. Apart from the mild elevation of ESR detected on the day of presentation with testicular ischaemia, all blood tests including CRP and SAA have remained steadfastly within the reference range before, during, and after testicular infarction (Fig. 4).

## Discussion and conclusions

We present the first description of vasculitic testicular infarction in a child with DADA2, thus expanding the phenotype even further. Our case highlights a number of important ongoing controversies and challenges regarding the management of DADA2, namely: lack of any biomarker to monitor patients for risk of vasculitic events, and hence when to initiate (arguably, lifelong) treatment with anti-TNF; controversies around the use of aspirin in DADA2; and lack of routine availability and reimbursement for anti-TNF treatment in patients with DADA2 in some countries.

DADA2 is heterogeneous in its severity with distinct lack of genotype/ phenotype correlation, and reports of adult patients with typical mutations and absence of ADA2 enzyme activity without any symptoms or signs. It has hitherto been assumed that presence of systemic inflammation would be an obvious indicator of need for treatment, and also other haematological and immunological manifestations such as marrow failure or immunodeficiency. This case illustrates that patients with absolutely normal laboratory parameters may also be at risk of developing acute ischaemic events, with apparently no obvious clinical triggers. Thus the negative predictive value for ischaemic events of CRP or SAA would be suggested to be low based on this case, since these were steadfastly within the normal reference range before, during, and after acute testicular infarction. We observed a transient rise of ESR on the day of tissue infarction, perhaps secondary to tissue injury. This indicates that conventional acute phase reactants are not necessarily predictive of disease activity and hence risk of severe events such as tissue infarction. In the future, this might be offered by peripheral blood cytokine analyses, peripheral blood cell transcription signals such as type I interferon regulated gene expression [15], recently suggested as a biomarker of disease activity in DADA2 [16], novel proteomics, or even some as yet undescribed biomarker derived from experience in other vasculitides such as circulating endothelial cells or endothelial microparticles [17–19].

Lack of a robust prognostic or disease activity biomarker predictive of adverse clinical outcomes makes therapeutic decisions for DADA2 difficult. One approach is to offer treatment with anti-TNF to all patients DADA2 upon diagnosis, even without any evidence of severe systemic vasculitis or autoinflammation. Arguably, in this case, this approach might have prevented testicular infarction. The downside is that this would result in initiation of expensive and lifelong immunosuppressive treatment for some patients who may never experience a clinical event, or who’s clinical event may be decades henceforth.

There has never been a randomised control therapeutic trial for DADA2, perhaps unsurprising since the disease was only first described in March 2014 [1, 2], and only approximately 200 cases are known about worldwide according to a recent patient registry curated by the DADA2 Foundation (http://www.dada2.org). Emphasising the rarity of the condition, and therefore the difficulties that would be encountered when considering performing a randomised controlled trial of frequentist statistical design. Consequently, anti-TNF is not routinely available for patients with DADA2 in the UK, even for those with severe symptoms.

Thus, countries which currently do not have policies for the routine prescribing and reimbursement of anti-TNF for patients with DADA2 need to consider an increasingly compelling “real-world” evidence in relation to the therapeutic benefit of anti-TNF in DADA2 particularly in relation to the prevention of stroke events. Recently, Ombrello et al. described outcomes in relation to anti-TNF treatment in their cohort of DADA2 patients at the National Institute of Health [12]. Pre-anti-TNF initiation, 55 strokes were observed in 15 patients over 2077 patient months. This fell to 0 stroke events over 733 patient months post anti-TNF initiation, a highly statistically significant finding clearly illustrating the benefit of anti-TNF for the prevention of strokes in DADA2 in a real-world setting. No hard data favour the use of one anti-TNF agent over another [3], although some physicians advocate the use of monoclonal antibodies against TNF rather than etanercept or etanercept-biosimilars, possibly based on suboptimal outcomes using etanercept in clinical trials for other vasculitides [20].

Two other important therapeutic points require some commentary. Our patient received corticosteroids and antiplatelet doses of aspirin. The rationale for corticosteroids to treat acute ischaemic events in DADA2 is to switch off vasculitis as quickly as possible, since the pathology is that of vascular occlusion secondary to vasculitis, often with platelet and endothelial activation in the context of systemic vasculitic inflammation, as previously described for other vasculitides [21–24]. Luminal arterial occlusion leading to reduced organ perfusion (Fig. 2), occurs from thrombotic occlusion, with or without a background of chronic luminal narrowing from vascular remodelling from chronic vasculitis, as seen in PAN [25]. Whilst the use of corticosteroids in DADA2 is based on experience gleaned from the treatment of other vasculitides [26–29], the additional therapeutic benefit of this approach versus anti-TNF alone is uncertain. Regarding aspirin, the rationale for this is again target the aforementioned platelet activation, with contribution to thrombus formation and luminal occlusion, as is the case for other vasculitides such as Kawasaki disease [30]. The particular concern of this approach, however, is that patients with DADA2 may present with haemorrhagic stroke (with or without thrombocytopenia) and thus aspirin would be contraindicated in that situation; or in the case of lacunar stroke, that the use of aspirin might result in haemorrhagic extension and clinical deterioration. In this case, with clear demonstration of acute testicular ischaemia and reduced perfusion, judicious use of anti aggregant dose of aspirin with careful monitoring for haemorrhagic extension seemed logical and safe, as borne out by the favourable clinical outcome.

Our case highlights the ever-expanding phenotype of DADA2, and highlights current therapeutic challenges. Improvements in our understanding of the pathogenesis will ultimately lead to better biomarkers, better treatments, and ultimately even cure using gene therapy based on the favourable clinical outcomes of patients undergoing allogeneic HSCT thus far.

## Conclusion

This is the first reported case of testicular ischaemia in the context of ADA-2 deficiency.

## Data Availability

This report contains anonymised data and material gleaned from routine clinical care and also ethically approved, research. No further sharing of clinical data is permitted since this would compromise patient privacy and anonymity.

## Abbreviations

ADA2: Adenosine deaminase 2
ANA: Antinuclear antibody
ANCA: Antineutrophil cytoplasmic antibody
Anti-TNF: Anti-tumour necrosis factor alpha
DADA2: Deficiency of adenosine deaminase 2
ESR: Erythrocyte sedimentation rate
HSCT: Haematopoietic stem cell transplantation
IgG: Immunoglobulin G
IL 6: Interleukin 6
SAA: Serum amyloid A
